# Increased Self-Efficacy among Older Adults Aging-in-Place during COVID-19

**DOI:** 10.1093/geroni/igab046.2351

**Published:** 2021-12-17

**Authors:** Lee Lindquist, Amber Miller, Ruqayyah Muhammed, Lauren Opsasnick, Alaine Murawski, Vanessa Ramirez-Zohfeld

**Affiliations:** 1 Northwestern University Feinberg School of Medicine, Chicago, Illinois, United States; 2 Northwestern University, Chicago, Illinois, United States; 3 Northwestern University Feinberg School of Medicine, Northwestern University Feinberg School of Medicine, Illinois, United States

## Abstract

Self-efficacy is defined as a person's belief in their capacity to execute behaviors necessary to produce specific performance attainments. It also reflects confidence in the ability to exert control over motivation, behavior, and environment. During the COVID-19 pandemic, older adults were stressed with forced isolation, concerns over mortality, and finding alternate means of fulfilling their home-based needs. We sought to assess how COVID-19 pandemic affected the self-efficacy of a cohort of older adults aging-in-place. The LITCOG cohort is a group of community-dwelling older adults (65 years and older) who have had longitudinally assessment of cognition, health literacy, and functional skills over the past 15 years. As part of a larger study of the LITCOG cohort assessing decision making for aging-in-place, we assessed self-efficacy using validated PROMIS (Patient-Reported Outcomes Measurement Information System) measures with older adults prior to COVID-19 and during the COVID-19 pandemic. Survey results were obtained from 214 subjects (n=66 pre-COVID and n=148 during COVID). Nearly half of the sample (48.2%) had either marginal (25.5%) or low health literacy (22.7%). PROMIS General Self Efficacy was higher among those assessed during the COVID-19 pandemic (45.8 (7.7) pre-COVID vs 43.7 (8.0), p=0.07). PROMIS Self Efficacy for managing social interactions was higher during the COVID pandemic (45.0 (6.1) pre-COVID-19 vs. 48.7 (8.3) during COVID-19, p=0.02). During the stress and social isolation of the COVID-19 pandemic, older adults exhibited increased levels of self-efficacy. Ongoing longitudinal follow-up will determine how this self-efficacy evolves after the COVID-19 pandemic and impacts the ability to age-in-place.

